# Immediate Prepectoral Breast Reconstruction in Suboptimal Patients Using an Air-filled Spacer

**DOI:** 10.1097/GOX.0000000000002470

**Published:** 2019-10-21

**Authors:** Hilton Becker, Prakash J. Mathew

**Affiliations:** From the * Cleveland Clinic Florida Department of Plastic Surgery Florida Atlantic University Charles E. Schmit College of Medicine,; Weston, Fla.; † Division of Plastic, Aesthetic, Reconstructive and Transgender Surgery, University of Miami Miller School of Medicine, Miami, Fla.

## Abstract

Immediate prepectoral breast reconstruction offers excellent aesthetic results with less pain and elimination of animation deformity due to avoidance of pectoralis dissection and subpectoral implant placement. Concerns about the effects of prostheses on flap perfusion have limited use of the technique to highly selected patients. We present a series of “suboptimal” patients that have undergone immediate prepectoral breast reconstruction utilizing an air-filled “spacer” implant.

**Methods:**

A single surgeon’s experience with immediate, single-stage prepectoral breast reconstruction using a Spectrum implant was retrospectively reviewed. Patient demographics, adjuvant therapies, risk factors for threatened flaps, and complications, including those that required subsequent intervention, were evaluated.

**Results:**

Twenty-five patients (39 breasts) underwent immediate prepectoral reconstruction with a Spectrum implant. Ten patients had minor complications, 6 of whom required intervention with successful correction. There was a single case of implant loss in the series; this patient had prior radiation.

**Conclusions:**

Utilizing the spacer concept, immediate single-stage prepectoral breast reconstruction is a viable alternative to subpectoral implant placement or delay procedures. The technique delivers aesthetic results with less postoperative pain, quicker operative times, and avoidance of animation deformity. It can be considered for any patient, including high-risk patients such as those with radiation exposure, thin/threatened skin flaps, significant ptosis, and obesity.

## INTRODUCTION

Two-stage subpectoral reconstruction remains the most common technique employed for alloplastic breast reconstruction after mastectomy.^1,2^ Although cited advantages include technical ease, reduced rates of capsular contracture and decreased infection rates, subpectoral placement is an invasive procedure—usually requiring ADM—that leads to increased pain, thinning of pectoralis muscle, and the dreaded “window shading” and “animation deformities.”^1,3–6^ There has been increased interest in prepectoral breast reconstruction, which offers a fast, easy way to perform alloplastic reconstruction while allowing for better contour, decreased pain, and lower cost.^4,7–9^ Prepectoral reconstruction can be done in a single stage using a gel-filled breast implant; however, it is often only performed on highly selected patients due to concerns about flap viability and perceived higher risk for flap necrosis. Patients deemed “high risk” with characteristics such as subjectively thin skin flaps, circulatory compromise, prior radiation, high BMI, and significant ptosis are subjected to delayed reconstruction along with the attendant risks and costs of a second procedure.^10^

The prepectoral approach is gaining popularity, and recent work has addressed appropriate patient selection particularly with regards to the “challenging patient.”^10^ We present a technique of prepectoral reconstruction that can be performed on virtually any patient. Immediate single-stage prepectoral reconstruction is performed using a nearly empty Spectrum adjustable implant (Mentor, Irvine, Calif.) that serves as a “spacer.” In this technique, no ADM is used which further reduces costs, operative time, and overall complication rate.^4,11^ The author’s consecutive series of suboptimal patients suggests that this technique can be applied to patients that were otherwise deemed too high risk for single stage or tissue expander-based prepectoral reconstruction.

## PATIENTS AND METHODS

A retrospective review was conducted on all patients who had undergone immediate single-stage breast reconstruction with a prepectoral spacer. All patients had spectrum implants placed, and none required placement of acellular dermal matrix. All patients who underwent this procedure were included in the analysis; there were no exclusion criteria. All procedures were performed by a single surgeon in a private practice setting. Informed consent for the procedure and photography was obtained for all patients. Patients were seen on the first postoperative day and frequently thereafter. Data collected included patient age, height, weight, diagnosis, history of chemotherapy and/or radiation, degree of ptosis, ancillary procedures performed, postoperative complications, and need for further intervention.

### Surgical Technique

The patient is marked preoperatively together with the breast oncologic surgeon. In smaller breasts, the inframammary incision is used. In patients for whom the nipple is to be excised or with significant ptosis, a vertical incision is used. In ptotic patients, no skin is excised. The skin is allowed to contract by keeping the implant underfilled.

At the completion of the mastectomy, the skin flaps and circulation are subjectively assessed by the reconstructive surgeon. Indocyanine green angiography is not used. Even if the skin flaps are borderline viable, the spacer that is placed is virtually empty and thus places no additional pressure on the mastectomy flaps.

After hemostasis is assured, the lateral skin flap is advanced medially and secured to the chest wall with interrupted 2-0 Vicryl (Ethicon, Inc., Somerville, N.J.). Up to 2 drains are placed through a long subcutaneous tunnel.

Based on the patient preference, volume of tissue resected, and the condition of the skin flaps, the appropriately sized Spectrum adjustable implant is selected. Only smooth round implants are used.^12^

**Fig. 1.**
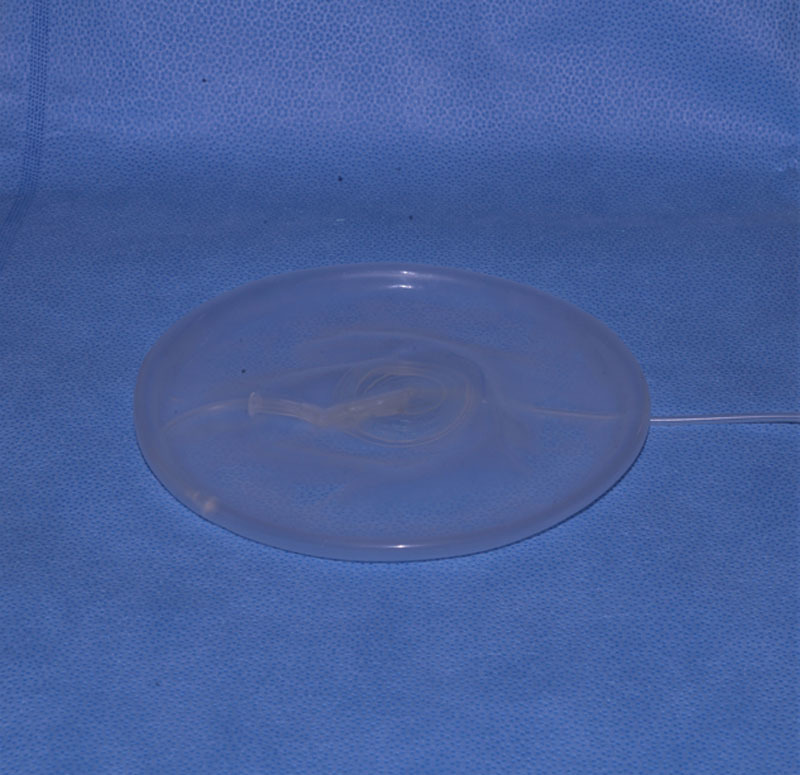
Empty Spectrum implant, which functions as a prepectoral spacer.

Air is evacuated from the implant save for a nominal volume that is retained to prevent implant collapse (Fig. 1). This use of air instead of saline for implant expansion is an off-label use of the device.^13–15^ The implant is placed in the pocket and closed, without the use of ADM or mesh.

The fill tube is cut to the appropriate length and attached to the injection port with the metal connector and 3-0 silk ties.

The injection port is then placed in a subcutaneous pocket and closed off with a 3-0 Vicryl (Ethicon, Inc., Somerville, N.J.) suture.

One or 2 drains are placed through long subcutaneous tunnels and secured to the skin. The pocket is irrigated with triple-antibiotic solution, and the incision closed with interrupted 3-0 Vicryl everting sutures and 2 rows of running 4-0 Monocryl sutures.^16^ A large *Tegaderm* Transparent Film Dressing (3M, St. Paul, Minn.) is applied to cover the entire breast.

The patient is assessed the next day. Once skin flap circulation is appropriate, further air is injected though a syringe filter (Cole-Parmer, Vernon Hills, Ill.) into the injection port.

**Fig. 2.**
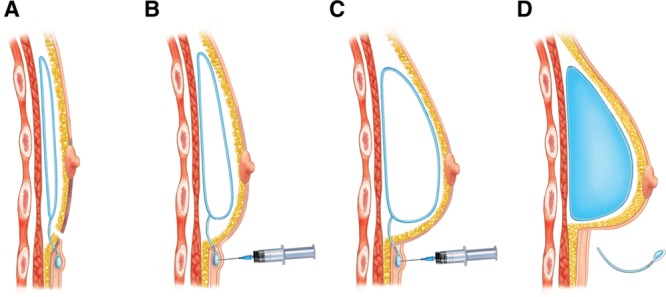
(A) An empty Spectrum implant is placed in the prepectoral position. (B) Once skin flap circulation is deemed appropriate, further air is injected through the subcutaneous fill port. (C) Serial injections of air are performed until the final desired size is achieved. (D) Air is replaced with saline and the injection port removed, leaving the patient with a permanent saline implant.

After achieving the correct position and shape, the air is replaced with saline, and the injection port is removed, leaving the patient with a permanent saline implant (Fig. 2). In countries where the adjustable gel saline implants are available, the result is a permanent gel saline implant. If there is significant ptosis, the breast is left underfilled with air for several weeks before replacing with saline to allow for sufficient contraction.^17^

Fat grafting is often performed to thicken the flaps. The saline implant can be exchanged for a gel implant in a second stage depending on patient preference or skin flap necessity. ADM can be used at this stage for further refinement.

## RESULTS

**Fig. 3.**
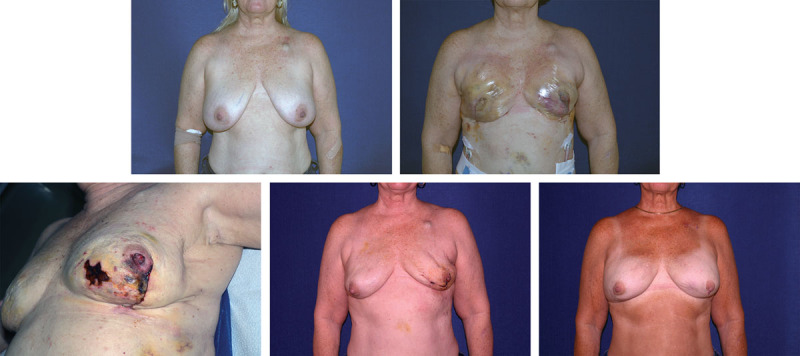
(A) 56-year-old woman with markedly ptotic breast and right breast carcinoma. (B) Early postoperative result. Flaps noted to be compromised. (C) Necrosis of flap. (D) Implant emptied, flap debrided and closed. (E) air removed and replaced with saline for final result.

**Fig. 4.**
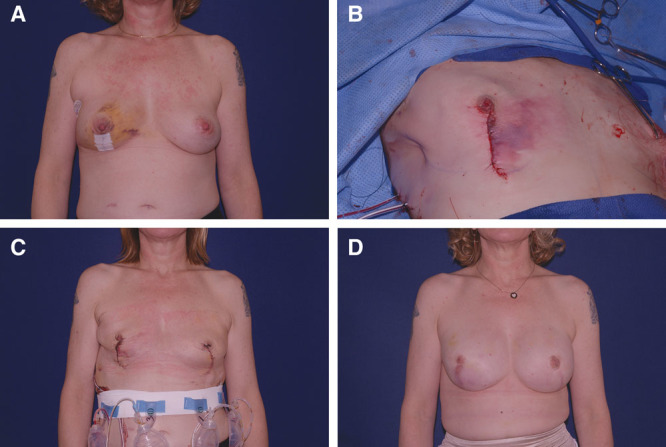
(A) 50-year-old woman with invasive ductal carcinoma of the right breast. (B) Skin closure after implant placement, circulation compromised. (C) Immediate postoperative result. (D) Final result following fat grafting.

**Fig. 5.**
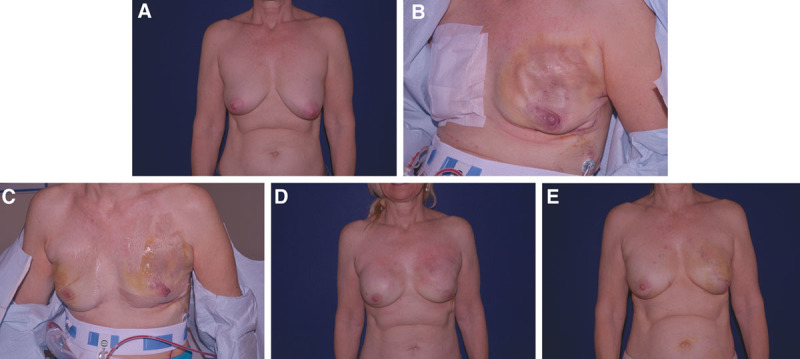
(A) 50-year-old patient with left breast carcinoma. (B) Circulation to skin flaps noted to be compromised intraoperatively so air was removed from the implant to relieve pressure. (C) Once circulation improved, air was added to the implant. (D) Once final size and shape was achieved, air was replaced with saline. (E) Implant was replaced with silicone gel implant.

Over a 1-year period, the author performed 25 consecutive cases of immediate prepectoral breast reconstruction using a spacer in suboptimal patients (Table 1; Figs. 3–5). Patient age ranged from 32 to 75 years, with a mean age of 53. BMI ranged from 19.4 to 37.8, with a mean of 26.0. Although the majority of operations were performed for active cancer diagnoses, the concept was also applied to prophylactic mastectomy (one patient) and operations for benign lesions (one patient). Nine patients underwent unilateral reconstruction, whereas 14 underwent bilateral reconstruction. Five patients (20%) were overweight with a BMI > 25. Three patients (12%) underwent adjuvant radiation, whereas 5 patients had a history of breast radiation before the procedure. Eight patients (32%) underwent a contralateral symmetry procedure, either reduction mammaplasty (3 patients) or mastopexy (5 patients). In terms of breast characteristics, 12 patients (48%) had some degree of ptosis, whereas 5 patients (20%) had large breasts. Thirteen patients (52%) underwent fat grafting.

**Table 1.** Patient Demographics, Intraoperative Flap Characteristics, and Results of Prepectoral Breast Reconstruction Using an Air-filled Spacer

Age (y)	BMI	Diagnosis	XRT +/−	Complication	Result	Obese +/−	Ptosis +/−	Large/Small	Flap
36	20.4	Paget’s—Right	+	−	−	−	+	−	−
59	28.8	BCA—right	+	−	−	+	−	−	−
73	21.1	BCA—left	−	−	−	−	+	Large	−
56	24.7	BCA—right	+	−	−	−	−	−	−
36	32.1	BCA—right	+	−	−	+	−	−	−
44	21.6	BCA—bilateral	−	Flap necrosis	Resolved	−	−	Small	Thin
71	32.6	BCA—left	−	Flap necrosis	Resolved	+	+	−	−
50	20.9	BCA—left	−	−	−	−	−	−	Thin
41	37.8	BCA—left	−	Flap necrosis	Resolved	+	+	Large	−
56	20.1	BCA—right	−	Flap necrosis	Resolved	−	+	−	−
74	26.6	BCA—right	−	−	−	+	+	Large	−
69	29.7	DCIS	−	−	−	+	+	−	−
40	20.5	DCIS—right	−	Flap necrosis	Resolved	−	−	Small	Thin
47	26.1	BCA—left	−	Seroma	Resolved	+	+	Large	−
53	19.4	BCA—left	−	Capsular contracture	Resolved	−	−	−	−
47	23.6	BCA—left	−	−	−	−	−	Small	Thin
43	22.1	BCA—right	+	−	−	−	−	−	−
41	24.2	BCA—left	−	−	−	−	−	−	−
59	33.1	BCA—left	+	Extrusion	Implant loss	−	−	−	−
32	26.6	Prophylactic	−	Flap necrosis	Resolved	+	+	−	−
69	22.8	BCA—left	−	Seroma	Resolved	−	+	−	−
57	22.1	BCA—right	−	−	−	−	−	−	−
52	26.6	BCA—left	−	−	−	+	+	Large	−
36	35.1	BCA—left	+	−	−	+	+	−	−
75	31.2	BCA—left	+	−	−	+	−	−	−

Ten patients had minor complications, and 6 required further intervention. Two patients developed seromas. One patient suffered capsular contracture. Five patients underwent successful debridement for skin flap edge flap necrosis with no further intervention. One patient who had prior radiation suffered extrusion of the implant that ultimately required removal. This was the only case of implant loss in the series.

## DISCUSSION

Breast cancer continues to be a leading cause of death in women.^18^ Breast oncologic surgery has advanced from the days of radical Halsted mastectomy to the current era of skin sparing and even nipple sparing mastectomy.^5,19,20^ Advances in reconstruction after ablative surgery have followed suit, and new techniques in alloplastic reconstruction afford women the opportunity for satisfactory aesthetic results without compromising oncologic safety.

Subpectoral reconstruction has demonstrated efficacy and, as a result, nearly 90% of all breast reconstruction performed in the United States employ this technique.^2^ Nonetheless, this technique not without risks, as there is potential for significant morbidity associated with manipulation of the pectoralis muscle. These include commonly cited issues such as animation deformity, window shading, thinning of pectoralis muscle with associated weakening of arm abduction, pain, and complications associated with performing another procedure in the 2-stage technique.^1,3–5,21,22^

Prepectoral reconstruction has several advantages when compared with the subpectoral technique. It is a technically easier procedure with decreased operative time.^2,8^ It also affords the ability to better define the breast contour.^23^ Previous work has also demonstrated that prepectoral reconstruction is cost effective, with a shorter average length of stay, quicker return to work, less narcotic use, less revisional surgery, and enhanced patient satisfaction.^11^ Despite manifold advantages, prepectoral reconstruction remains an underutilized technique that is only offered to highly selected patients.

In our technique, prepectoral reconstruction is performed in all patients in a single stage, utilizing the concept of a “spacer” for high-risk patients. In these patients, the spacer prevents pressure from being applied to the skin flaps, allowing for circulation to recover before the implant is filled. The spacer affords mastectomy flaps to recover from ischemia in a manner similar to performing delayed reconstruction.

Patients generally deemed suboptimal for prepectoral reconstruction include patients with larger breasts, as these patients would require larger implants that could cause circulatory compromise in the skin flaps. Similarly, patients with significant ptosis are excluded from single-stage procedures as repositioning of the nipple-areola complex at the apex of the breast mound may lead to ischemic compromise.^10^ Patients that have undergone radiation either preoperatively or in an adjuvant setting are also at higher risk for complications.^24^ Our spacer technique allows for these skin flaps to recover before being filled to the appropriate size.

Cost containment remains an important consideration in healthcare worldwide. Our technique offers several opportunities for cost savings throughout the perioperative period. Intraoperatively, the technique allows for decreased operative time and avoids the use of ADM or indocyanine green angiography, both of which add significant cost to the operation.^25–28^ Postoperatively, patients have decreased narcotic usage, decreased length of stay, and quicker return to work. They are also subjected to less expansions and are able to avoid a second-stage operation.

The most important factor to be considered when performing immediate prepectoral breast reconstruction is the quality and circulation of the skin flaps. Unfavorable skin flaps are seen as a relative contraindication to a single-stage procedure, or even tissue expander reconstruction due to the bulk of the prostheses. Our technique utilizes a virtually empty spacer that allows suboptimal skin flaps to recover circulation and avoid ischemic complications.

## CONCLUSIONS

Alloplastic breast reconstruction continues to evolve, and there is renewed interest in prepectoral breast reconstruction. Single-stage prepectoral breast reconstruction is a viable technique with considerable advantages. Our technique allows prepectoral reconstruction to be performed on patients that are considered “suboptimal” and would otherwise not be candidates for immediate prepectoral reconstruction. Although further ongoing long-term follow-up is required, our data suggest that this technique is safe for virtually any patient.
